# Comparative study of the nutritional composition of local brown rice, maize (obaatanpa), and millet—A baseline research for varietal complementary feeding

**DOI:** 10.1002/fsn3.1556

**Published:** 2020-04-14

**Authors:** Nancy Yankah, Freda Dzifa Intiful, Edem M. A. Tette

**Affiliations:** ^1^ Department of Nutrition and Dietetics School of Biomedical and Allied Health Sciences College of Health sciences University of Ghana Accra Ghana; ^2^ Department of Community Health School of Public Health College of Health Sciences University of Ghana Accra Ghana

**Keywords:** brown rice, complementary feeding, maize, millet

## Abstract

**Introduction:**

Childhood malnutrition remains a major public health issue of concern particularly in sub‐Saharan Africa, and inadequate complementary feeding is a common cause. Promoting dietary diversity is one way of tackling this problem. High dependence on maize has its limitations; modifying other local staples into complementary foods can be a feasible alternative to promote optimum nutrition.

**Objectives:**

Comparing the nutritional composition of brown rice to millet and maize to determine its beneficial value as complementary food.

**Methods:**

Experimental study was carried out at the Department of Nutrition and Food Science of University of Ghana. Samples of maize, millet, and brown rice were obtained from the Ministry of Agriculture, Accra and nutritional contents analyzed. Statistical Package for Social Sciences version 20.0 and ANOVA were used to assess differences.

**Results:**

Results showed brown rice contained the highest content of carbohydrates (77.94 ± 0.32) % and zinc (12.15 ± 0.21) mg while millet had the highest protein (10.49 ± 0E‐7) mg and fat (4.99 ± 0.46) % content. Maize contained highest amount of calcium (21.24 ± 0.14) mg. Iron was only found in millet (10.72 ± 0.15) mg. The zinc content per 100 g of all three (3) cereals was above RDA. All three (3) cereals contributed significantly <10% to the RDA of calcium. Iron content of millet contributed more than 90% to RDA.

**Conclusions:**

Locally produced brown rice is rich in zinc and carbohydrates compared to millet and maize. Thus, can be used for complementary feed but, given the low protein and iron content, it may need to be fortified or diversified and used as a cereal blend.

## Graphical abstract

Diversity in complementary feeding improves its nutritional quality. Locally produced brown rice is rich in micronutrients such as zinc compared to maize and millet. It can be a suitable alternative for use as complementary feed; however, its protein and iron contents can be improved with addition of legumes.

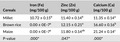

## 1 INTRODUCTION

In spite of efforts to curb malnutrition, recent estimates indicate that the global stunting, wasting, and severe wasting rates are 21.9%, 7.3%, and 2%, respectively (UNICEF, 2019). In Africa, the prevalence of stunting is reported to be about 33%. Although the levels of stunting in under five‐year‐olds in Ghana has dropped, from 28% (GDHS, 2008) to 19% (GHS 2014), Ghana still faces a major challenge with childhood malnutrition including micronutrient malnutrition which has been reported to be highly prevalent and persistent with anemia in 66% of children <5 years old (GSS, 2015). Inappropriate introduction of complementary feeding is a major risk factor, and the incidence of malnutrition tends to rise with the introduction these foods (Lassi, Zahid, Das, & Bhutta, 2013; Michaelsen, Grummer‐Strawn, & Bégin, 2017; Tette, Sifah, Tete‐Donkor, Nuro‐Ameyaw, & Nartey, 2016). According to Bhutta et al. (2013), inappropriate complementary feeding accounts for about 100,000 of deaths among children below five years old and some consequences of inadequate nutrition during the first 1,000 days of a child's life are very difficult to reverse (Schwarzenberg & Georgieff, 2018). Thus Affordable, appropriate and timely introduction of complementary foods is a key to maintaining nutritional status (WHO, 2002).

The nutritional demands of infants necessitate the introduction of complementary foods in a safe and timely manner after exclusive breastfeeding for the first 6 months of life while continuing breastfeeding because breast milk alone cannot meet nutritional needs during this period (Lassi et al., 2013; PAHO, 2003). In most sub‐Saharan African countries, complementary foods are mainly cereal based. In Ghana, whole grains of maize and millet are commonly used (Suri, Tano‐Debrah, Ghosh, 2014). The most favoured foods are fermented corn dough porridge (*Koko*), millet porridge (*hausa koko*), and roasted corn porridge (*Tom‐brown*) (Colecraft et al., 2004). Although these whole grains serve as sources of nutrients such as carbohydrates, fiber and the B vitamins (Abeshu, Lelisa, & Geleta, 2016), research has shown that *Koko* and *Tom‐brown* are poor in protein quality, have low energy density, and do not support the growth of some children adequately (Colecraft et al., 2004). Another common weaning food is *weanimix*, a cereal‐legume blend, which is bulky and hence refused by some children. This has created a need to explore the nutritional composition of other indigenous staples to seek alternatives.

Ensuring dietary diversity is one of the key ways to improving dietary quality of complementary feeds (Ogunba, 2010). Increasing diversity of the diet is an effective way of improving its quality and meeting micronutrient requirements (Daelmans et al., 2009). Brown rice is a staple food for the people of the Volta region of Ghana especially at Avatime where they are known to celebrate a Brown Rice Festival. However, according to a report by Badi (2013), locally available brown rice is patronized by only a few in the country although this can be diversified and used as a complementary food. Even though locally produced brown rice is poorly patronized by consumers in Ghana, majority of imported complementary foods are rice based with their prices ever escalating. These hikes in prices cause parents to dilute these complementary foods in other to avoid its financial repercussions on the family thereby resulting in malnutrition. Most malnutrition cases reported in pediatric wards are due to over dilution of commercial foods. Indigenous brown rice on the other hand is cheaper and readily available as compared to imported complementary foods. There is lack of data on the nutritive value and suitability of brown rice as an alternative cereal for complementary feeding. Therefore, the aim of this study was to compare the nutritional composition of local brown rice (*Oryza glaberrima*), millet (*Pennisetum glaucum*), and maize (*Zea mays*) to determine its beneficial value as a complementary food.

## 2 METHODOLOGY

### 2.1 Study design/site

An experimental study design was employed. Samples of the cereals were obtained from the Ministry of Agriculture shop located in Accra which sells food products from the regions. The experimental analysis was carried out at the food analysis laboratory of the Department of Nutrition and Food Science, University of Ghana.

### 2.2 Sampling

One kilogram each of brown rice (*Oryza glaberrima*), Pearl millet (*Pennisetum glaucum*), and obaatanpa maize (*Zea mays*) were purchased from the study site. Two samples were obtained from each of the cereal varieties and analyzed in the laboratory. The process of quartering was used in obtaining the samples for analysis.

### 2.3 Quality control and assurance

All samples were obtained from the Ministry of Agriculture shop because it is an outlet that ensures the marketing of standard quality produces from farmers across the country. Additionally, it was ensured that the batch selected for analysis was not insect infested, did not contain foreign particles, and was considered wholesome based on the Ministry of Agriculture shop outlet criteria.

### 2.4 Laboratory analysis

Duplicate samples of each of the purchased cereals were analyzed using standard method of analysis of nutrients in foods as follows:

#### 2.4.1 Protein

The protein content of each sample was determined by following standard procedure of the Kjedahl method (AOAC Official Method 984.13A, 2006).

#### 2.4.2 Ash

The ash content was determined by incinerating with furnace at 550°C (method 932‐03, AOAC, 1990).

#### 2.4.3 Crude fat

The Soxhlet extraction method was used in the determination of total crude fat content of the (AOAC Official method 920.39c, 2006).

#### 2.4.4 Moisture

The hot hair oven method was used to determine the percentage of moisture in the selected cereals (AOAC Official Method934.01, 2006).

#### 2.4.5 Carbohydrate

The difference method was adopted to calculate total carbohydrates by subtracting the percentage of moisture, protein, fat, and ash contents from 100% (AOAC Official method954.11, 2000).

#### 2.4.6 Iron

Iron content was determined by Atomic Absorption method (AAS) (AOAC Official method 999.10b, 2000).

#### 2.4.7 Zinc

Zinc content was determined by Atomic Absorption method (AAS) (AOAC Official method 999.10b, 2000).

#### 2.4.8 Calcium

Calcium content was determined by Atomic Absorption method (AAS) (AOAC Official method 999.10b, 2000).

### 2.5 Data analysis

Data entry and analysis were done using the software Statistical Program for Social Sciences (SPSS) version 20.0. Means and standard deviations were used to describe the spread of the data. Analysis of variance (ANOVA) was used to compare differences between the means of the nutrients in the different varieties of cereals. Statistical significance was set at *p* < .05.

## 3 RESULTS

Table 1 describes the average percent content of carbohydrate, protein, fat, ash, and moisture of the cereals. The fat content in millet was found to be significantly higher than maize (*p* = .015). Similarly, the protein content was significantly higher than millet and brown rice (*p* < .0001). Brown rice had the highest composition of carbohydrates (*p* = .004). There were no significant differences between the moisture contents of the cereals. Ash content was highest in millet compared to brown rice and maize (*p* = .001).

**Table 1:** Percent moisture, ash, fat, protein, and carbohydrate contents of cereals

Cereal	Fat (%)	Protein (%)	Carbohydrate (%)	Moisture (%)	Ash (%)
Millet	4.99 ± 0.46^a^	10.49 ± 0E−7^a^	70.41 ± 1.03^a^	12.64 ± 1.53	1.46 ± 0.03^a^
Brown rice	4.67 ± 0.01^a^	4.28 ± 0.19^b^	77.94 ± 0.32^b^	12.31 ± 0.14	0.79 ± 0.00^b^
Maize	3.28 ± 0.01^b^	8.90 ± 0.16^c^	73.94 ± 0.51^c^	13.07 ± 0.41	0.79 ± 0.07^b^
*p*‐value	.015*	.0001*	.004*	.729	.001*

Note

Tukey's post hoc significant at ^*^ *p* ≤ .05.

Composition of three selected minerals in the cereals is presented in Table 2. There were significantly higher levels of iron in millet compared to brown rice and maize which reported undetectable amounts. Brown rice contained significantly higher content of zinc compared to millet. The amount of calcium in maize was found to be higher than that in millet and brown rice.

**Table 2:** Composition of iron, zinc, and calcium in cereals

Cereals	Iron (Fe) (mg/100 g)	Zinc (Zn) (mg/100 g)	Calcium (Ca) (mg/100 g)
Millet	10.72 ± 0.15^b^	11.40 ± 0.14^b^	11.35 ± 0.14^a^
Brown rice	0.00 ± 0E−7^a^	12.15 ± 0.21^a^	16.60 ± 0.16^b^
Maize	0.00 ± 0E−7^a^	11.80 ± 0.14^ab^	21.24 ± 0.14^c^
P‐value	.000*	.047*	.000*

Note

Tukey's post hoc significant at ^*^ *p* ≤ .05.

Tables 3 and 4 report on a comparison of the micronutrients (iron, zinc, and calcium) content with the recommended daily allowance of infants 7–11 months and 1‐ to 3‐year‐olds, respectively. The results indicate that maize and brown rice contributed 0% of iron to the RDA. However, the content of zinc in all three varieties contributes over 300% of the RDA. The calcium in maize provided the highest contribution to RDA compared to millet and brown rice.

**Table 3:** Comparison of micronutrient (iron, zinc, and calcium) content with recommended daily allowance of infants (7–11 months)

Cereal	Minerals	mg/100 g	RDA	%RDA met
Maize	Iron	Below detection	11	0
	Zinc	11.70	3	390.00
	Calcium	21.40	260	8.23
Brown rice	Iron	Below detection	11	0
	Zinc	12.00	3	400.00
	Calcium	16.48	260	6.34
Millet	Iron	10.61	11	96.45
	Zinc	11.30	3	376.67
	Calcium	11.25	260	4.33

**Table 4:** Comparison of micronutrient (iron, zinc, and calcium) content of cereals to RDA of infants (1–3 years)

Cereal	Minerals	mg/100 g	RDA (mg/100 g)	%RDA
Maize	Iron	Below detection	7	
	Zinc	11.70	3	390.00
	Calcium	21.40	700	3.06
Brown rice	Iron	Below detection	7	0
	Zinc	12.00	3	400.00
	Calcium	16.48	700	2.35
Millet	Iron	10.61	7	151.57
	Zinc	11.30	3	376.67
	Calcium	11.25	700	1.61

## 4 DISCUSSIONS

### 4.1 Moisture

No matter the intended purpose of a particular cereal, its moisture content has significant implications on its quality (Wilkin & Stenning, 1989). According to Kumar et al., (2016), the chemical analysis of brown rice showed a moisture content of 12.4%. This is similar to what was obtained in this study. They also determined the moisture content of millet and maize to be 12.4% and 10.4%, respectively. The differences in moisture contents could be attributed to storage conditions. Poor storage of cereals such as storing in thick sacks on bare floor and warm rooms can affect the moisture content of stored cereals. Saleh at al. (2013) determined moisture content of brown rice, millet, and maize to be 12.0% for all the three cereals. This moisture content is significantly different compared to that of the cereals in this present study. The present study also showed that the moisture content of all three cereals analyzed was below 14%. This level of moisture ensures that little metabolic activity occurs thereby preserving the cereal from deteriorating quickly (Wilkin & Stenning, 1989).

### 4.2 Ash

In general terms, the ash content of a particular product is a representation of the minerals present in the product (Ahmed, Shoaib, Akhtar, & Iqbal, 2014). According to Saleh et al. (2013), the chemical analysis of brown rice had an ash content of 1.3% and that of locally produced cereals contained 0.79%. They also determined the ash content of millet and maize to be 2.2% and 1.2%, respectively. The ash content in millet and maize analyzed in the present study was 1.46% and 0.79%, respectively. The differences in percentages could be due to varietal differences as well as environmental conditions (Ahmed et al., 2014).

In another study conducted by Kumar et al., (2016) the ash content of brown rice and maize was not recorded; however, ash content of millet was 2.3%. Comparing the ash content of locally produced millet which contained 1.46%, it shows a wide difference and this can be attributed to the variety of millet used.

### 4.3 Carbohydrates

Carbohydrates are one of the main source of energy to the body. Carbohydrates content for brown rice, millet, and maize according to Kumar et al., (2016) was 76.2%, 67.5%, and 74.3%, respectively. Additionally, Saleh et al., (2013), recorded carbohydrates content for brown rice, millet, and maize to be 76.0%, 67.0%, and 73.0%, respectively. These were comparable to the contents of carbohydrates in three cereals analyzed in this study. This shows that the locally produced cereals are very high in carbohydrates and similar to the values determined by Kumar et al. (2016) and Saleh et al. (2013). Furthermore, a comparative study conducted by Eggum (1979) showed that carbohydrates content for brown rice, millet, and maize was 74.8%, 73.7%, and 74.0%, respectively. There was significant difference in the carbohydrates content of brown rice in this study compared to that of Eggum (1979). This difference can be attributed to the variety of brown rice and also different agricultural factors. Millet in the present study recorded lower carbohydrate content and can also be attributed to varietal difference. Maize had almost the same values.

### 4.4 Protein

Proteins represent polymers of amino acids in a particular product. The amount of amino acids present is indicative of the quality of the protein (Ahmed eta al., 2014). Protein content as analyzed by Kumar et al. (2016) was 7.5%, 11.6%, and 9.4% for brown rice, millet, and maize, respectively. Saleh et al. (2013) also analyzed protein content to be 7.9%, 11.8%, and 9.2%, respectively, for brown rice, millet, and maize. In this study, the protein content was found to be lower in all the cereals as compared to the earlier studies. The differences in nutrient contents could also be attributed to varietal differences.

Brown rice had significantly low content of protein also that of millet and maize were lower than already determined values. Protein content as analyzed by Eggum (1979) was 8.5%, 13.4%, and 11.4% for brown rice, millet, and maize, respectively. Generally, the protein content of the cereals was lower compared to that of already determined values. The reasons for this disparity can be explained by the likely difference in variety, the method of analysis as some of the cited works used different procedures in determination of protein content. Also, the different moisture content may also have accounted for the differences recorded. In addition, Ahmed et al. (2014) asserts that processing could also affect the protein content in a cereal.

### 4.5 Fat

Fats are the main source of energy in foods and also provide characteristics such as flavor, taste, texture, and appearance to food (Ahmed et al., 2014). Fat content of brown rice, millet, and maize using the same analytical method was rice 2.7%, 5%, and 4.7%, respectively, according to Kumar et al. (2016). The brown rice analyzed in this study had a fat content of 4.67%. The difference in fat composition could be attributed to varietal differences, mode of preservation, and different methods of processing (Ahmed et al., 2014). In India, Mudos and Das (2018) reported fat composition in brown rice to range between 1.95% and 3.83%. Their study confirmed an already established finding that traditional rice varieties had higher fat composition compared to imported varieties. The fat tends to improve upon the taste of rice. Millet analyzed had a fat content of 4.99% which is not too different from other analysis done. This study will help increase the use of brown rice as a complementary food. This information also has the potential to increase recognition and patronage for locally produced brown rice, creating revenue for cereal farmers after dissemination. Fat content of maize was determined to be 3.28% which also showed significant difference compared to 4.7% from Kumar et al. (2016).

In another study done by Saleh et al. (2013) which analyzed the nutritive value of millet together with other grains, it was found that the fat content of brown rice, millet, and maize was 2.7%, 4.8%, and 4.6%, respectively. The millet analyzed in the present study had a fat content which is not too different from studies done in other countries. Fat content of maize in this study was found to be lower compared to that reported by Saleh et al. (2013). The difference in fat composition can be explained by high moisture content determined and the varietal difference in maize.

Fat content of brown rice, millet, and maize using the same analytical method was 2.6%, 5.5%, and 5.7%, respectively, according to Eggum (1979). The brown rice analyzed had a fat content of 4.67%; this shows that locally produced brown rice is high in fat compared to already determined values. Fat content of maize was determined to be 3.28% which also shows significant difference; this can be due to the high moisture content determined and the varietal difference in maize. On the average, there was a slight difference in the fat contents of cereals analyzed and that of already determined values; this may be due to calibration differences.

### 4.6 Iron

Iron is one of the nutrients that is of utmost public health importance. Its deficiency is very high among developing children, and therefore, a good complementary food should have adequate amounts of iron. A comparative analysis performed by Kumar et al. (2016) showed that brown rice contained 1.8 mg of iron and maize 2.7 mg. According to the same study, millet contained 8 mg of iron whiles in the present study millet contained 10.72 mg of iron. However, in this study, iron content of maize and brown rice was below detectable ranges. Saleh et al. (2013) also showed that brown rice contained 1.8 mg of iron per 100 g and millet 11 mg per 100 g. Additionally, in a study by Eggum (1979), the iron content of brown rice was shown to contain 3.0 mg iron per 100 g and maize contained 4.0 mg of iron per 100 g. Contrary to their findings, the iron content of maize and brown rice in this study was below detectable ranges. This is unusual as no study has reported undetectable levels. Notwithstanding, it must be emphasized that the method used in assessing the iron content is only capable of determining iron content when concentrations in the product are above 7 mg/kg (Jorhem & Engman, 2000), therefore, indicating that the iron content was below detectable levels does not imply the absence of iron in the cereals. However, a myriad of factors can be attributed to this finding. This may include varietal differences, extremely low iron content in the soil from which the grains were planted, as well as grain processing and preservation procedures carried on the grains. Nevertheless, this can be improved through fertilization application, agronomic bio‐fortification, and genetic engineering (Bilski, Jacob, Soumaila, Kraft, & Farnsworth, 2012; Gregorio, Senadhira, & Htut, 1999).

### 4.7 Zinc

Zinc as a nutrient is vital for cell growth, metabolism, and differentiation. Its deficiency results in restricted growth in childhood, decreased resistance to diseases and infections, and significantly contributes to mortality and morbidity (Darnton‐Hill, 2013). Kumar et al., (2016), determined the zinc content of brown rice and millet to be 2.02 and 3.0 mg per 100 g, respectively, whereas Eggum (1979) reported 3.0, 2.0, and 3.0 mg in maize, brown rice, and millet, respectively. Across a range of cereals and pulses, Hemalatha, Platel, and Srinivasan (2007) reported zinc content to be between 1.08 and 2.24 mg per 100 g. However, in this study, zinc content ranged between 11.3 and 12 mg. This is an indication of very high content of zinc in the products analyzed. Zinc deficiency still remains a public health issue in Ghana (Egbi, 2012). Therefore, the need to enrich crops with zinc is imperative. Hence, the high zinc content observed could be attributed to improvement in the crop varieties through agricultural mechanisms. Mechanisms such as fertilizer application to the soil and genetic engineering have aided in improving the zinc concentration of cereals to over 60% in some countries (de Valença, Bake, Brouwer, & Giller, 2017).

### 4.8 Calcium

Calcium is a mineral which is crucial in bone development particularly during infancy and childhood. According to Kumar et al., (2016), the content of calcium in 100 g of brown rice was analyzed to be 33 mg. The calcium content in brown rice in the present study was 16.48 mg, an indication that the locally produced brown rice is low in calcium compared to the other varieties from other countries. According to Kumar et al., (2016), millet contained 42 mg of calcium. In the present study, millet contained 11.25 mg of calcium, and this shows that calcium content of present work is lower compared to already analyzed millet. Maize according to Kumar et al., (2016) has 7 mg; calcium content of locally analyzed maize was 21.14 mg. Calcium content in maize in the present study was very high compared to that of Kumar et al., (2016).

Saleh et al. (2013) showed that brown rice contained 33 mg of calcium per 100 g. That of the locally produced brown rice analyzed was 16.48 mg this shows the calcium content of locally produced brown rice differs from that of already conducted analysis. In the same work, millet contained 42 mg of calcium that of the present product analyzed millet contained 11.25 mg per 100g which is much lower than what was determined in Saleh et al. (2013) work. Maize according to Saleh et al. (2013) has 26 mg of calcium. Locally produced maize contained 21.14 mg of calcium. Calcium content of locally produced maize is lower compared to already determined values, and this may be due to varietal differences. In summary, calcium content of cereals analyzed was lower compared to that of already determined values.

In Ghana, maize is commonly used as a complementary food due to its availability and cost. Though there are several varieties of Maize, *Obaatanpa* maize variety, which was used in this study, is the most commonly used for complementary feeding as it is recommended by the Ministry of Agriculture due to the perception that it has high content of protein. However, it is actually lower in protein compared to other varieties from other studies. From the analysis, all three (3) cereals are rich in one nutrient or the other. For instance, for the micronutrients analyzed each cereal recorded the highest in one of them; brown rice was highest in zinc whereas millet and maize were highest in iron and calcium, respectively.

The absorption of minerals in cereals by the body can, however, be inhibited by phytates which are also components of cereals. This study did not look at the phytates composition in the cereals analyzed, and therefore, the molar ratios of minerals to phytates cannot be ascertained. Nonetheless, comparison with RDA provides an idea of how much nutrients can be found in the cereals. It should be noted that bioavailability of minerals can be affected as a result of the amount of phytates present in the cereals.

Dietary diversity is essential; hence, incorporating these three (3) cereals in a child's diet will provide an array of nutrients essential for growth. Furthermore, these cereals are not consumed in their raw state; their preparation processes can affect the availability of nutritional content. Nonetheless, sprouting can be used as an important tool to improve the quality of cereals (Mbithi‐Mwikya, Camp, Yiru, & Huyghebaert, 2000). Sprouting was found to improve nutrients such as essential amino acids, B vitamins and also increases digestibility of cereals. Enriching these cereals with breast milk and infant milk will improve their nutritional content and also by the addition of legumes in their right proportions (Olukemi Samuel & Omolara Otegbayo, 2006). In addition, cereals such as brown rice and maize can be fortified with iron and calcium to boost their nutritional content.

## 5 CONCLUSION

This study showed that brown rice contained the highest content of carbohydrates, while millet had the highest in protein and fat content. The level of zinc determined was highest in brown rice with millet recording the lowest content. There was a relatively high amount of calcium in maize. Apart from millet which recorded substantial amount of iron, the rest were below detectable range. The zinc content per 100 g of all three (3) cereals was above RDA. All three cereals contributed significantly less than 10% to the RDA of calcium. Iron content of millet contributed more than 90% to RDA per 100 g. Locally produced brown rice is rich in zinc and carbohydrates compared to millet and maize. Thus can be used for complementary feed but, given the low protein and iron content, it may need to be fortified or diversified by blending it with a legume.

## CONFLICT OF INTEREST

None declared.

## AUTHOR CONTRIBUTION

EMAT and FDI conceived idea; NY collected and analyzed data; FDI, NY, and EMAT wrote and approved manuscript.

## ETHICAL APPROVAL

The protocol was reviewed by the ethics and protocol review committee of the School of Biomedical and Allied Health Sciences.
